# Flattened Structural Network Changes and Association of Hyperconnectivity With Symptom Severity in 2–7-Year-Old Children With Autism

**DOI:** 10.3389/fnins.2021.757838

**Published:** 2022-02-14

**Authors:** Minhui Ouyang, Yun Peng, Susan Sotardi, Di Hu, Tianjia Zhu, Hua Cheng, Hao Huang

**Affiliations:** ^1^Department of Radiology, Children’s Hospital of Philadelphia, Philadelphia, PA, United States; ^2^Department of Radiology, Perelman School of Medicine, University of Pennsylvania, Philadelphia, PA, United States; ^3^Department of Radiology, Beijing Children’s Hospital, Capital Medical University, Beijing, China; ^4^Department of Bioengineering, School of Engineering and Applied Science, University of Pennsylvania, Philadelphia, PA, United States

**Keywords:** autism spectrum disorder, brain development, structural network, hyper-connectivity, early childhood, symptom severity

## Abstract

Understanding the brain differences present at the earliest possible diagnostic age for autism spectrum disorder (ASD) is crucial for delineating the underlying neuropathology of the disorder. However, knowledge of brain structural network changes in the early important developmental period between 2 and 7 years of age is limited in children with ASD. In this study, we aimed to fill the knowledge gap by characterizing age-related brain structural network changes in ASD from 2 to 7 years of age, and identify sensitive network-based imaging biomarkers that are significantly correlated with the symptom severity. Diffusion MRI was acquired in 30 children with ASD and 21 typically developmental (TD) children. With diffusion MRI and quantified clinical assessment, we conducted network-based analysis and correlation between graph-theory-based measurements and symptom severity. Significant age-by-group interaction was found in global network measures and nodal efficiencies during the developmental period of 2–7 years old. Compared with significant age-related growth of the structural network in TD, relatively flattened maturational trends were observed in ASD. Hyper-connectivity in the structural network with higher global efficiency, global network strength, and nodal efficiency were observed in children with ASD. Network edge strength in ASD also demonstrated hyper-connectivity in widespread anatomical connections, including those in default-mode, frontoparietal, and sensorimotor networks. Importantly, identified higher nodal efficiencies and higher network edge strengths were significantly correlated with symptom severity in ASD. Collectively, structural networks in ASD during this early developmental period of 2–7 years of age are characterized by hyper-connectivity and slower maturation, with aberrant hyper-connectivity significantly correlated with symptom severity. These aberrant network measures may serve as imaging biomarkers for ASD from 2 to 7 years of age.

## Introduction

Autism spectrum disorder (ASD) is characterized by deficits in social communication and interactions, repetitive patterns of behaviors, and restricted interests (Constantino and Charman, 2016). The age range of 2–7 years is a critical developmental period for understanding ASD as it is a time frame immediately after the earliest possible ASD diagnosis around 2–3 years of age based on current clinical diagnostic methods (Ozonoff et al., 2010). However, nearly all the current literature on brain structure and function in ASD focuses on late childhood periods after 7 years of age (e.g., Keown et al., 2013; Supekar et al., 2013; Payabvash et al., 2019), or adolescent and adult groups (e.g., Rudie et al., 2013; Di Martino et al., 2014; Nomi and Uddin, 2015). Recently, a few retrospective neuroimaging studies included very young children before 2–3 years who went on to be diagnosed with ASD (Levman et al., 2018, 2019; Shiohama et al., 2021). Diffusion MRI studies on the important early developmental period of 2–7 years in ASD are relatively scarce and were conducted to investigate white matter microstructural changes (e.g., Ben Bashat et al., 2007; Walker et al., 2012; Ouyang et al., 2016). Since ASD has numerous implications for patient function and integration in society, early interventions for ASD immediately after its diagnosis at around 2 years of age are more likely to reduce symptoms and to positively affect long-term neurodevelopment (Zwaigenbaum et al., 2015). Current lack of understanding about ASD in the age range of 2–7 years limits potential approaches for early intervention.

Connectomics, a framework to comprehensively map brain organization into a network (Bullmore and Sporns, 2009), has been applied to studying a variety of brain disorders (van den Heuvel and Sporns, 2019). Topological properties of complex brain networks can be quantified with graph theoretical analysis. The network is represented as nodes of a graph with connecting edges based on measures of connectivity. A growing body of literature suggests that ASD is associated with alterations in multiple interconnected brain systems rather than isolated regions (e.g., Minshew and Williams, 2007; Rudie et al., 2013; Uddin et al., 2013; Nomi and Uddin, 2015). Meanwhile, large-scale brain systems affected in ASD may underlie patients’ complex phenotype of behavioral impairments. For instance, prior studies reported that the aberrant brain connectivity of the default mode network (DMN) is likely linked to social deficits in ASD (e.g., Assaf et al., 2010; Lynch et al., 2013). Using connectome analysis to systematically delineate alterations in brain organization of ASD may contribute to understanding its underlying neuropathology and identifying sensitive network-based imaging biomarkers that are correlated with its symptom severity.

Heterogenous patterns of aberrant brain connectivity, characterized by both hyper- and hypo-connectivity, have been reported in prior studies on ASD (e.g., Keown et al., 2013; Rudie et al., 2013; Supekar et al., 2013; Uddin et al., 2013; Di Martino et al., 2014; Li et al., 2014; Nomi and Uddin, 2015). Specifically, brain hyper-connectivity is more common in children with ASD, while hypo-connectivity is prevalent in adolescents and adults with ASD compared to typically developing (TD) individuals (Keown et al., 2013; Supekar et al., 2013; Uddin et al., 2013). The discrepancies between findings of hyper- and hypo-connectivity are likely dependent on altered age-related trajectories associated with ASD in different developmental stages (e.g., Uddin et al., 2013; Ouyang et al., 2017). With connectomic analysis as an effective tool to probe brain organizational changes, characterizing age-related brain network and connectivity changes in ASD in different developmental stages may elucidate the non-uniform changes associated with ASD. Structural networks, usually constructed by white matter (WM) bundles traced with diffusion MRI (dMRI) tractography, serve as the structural substrate of brain functional organization. So far, the majority of the network and connectivity research in ASD has adopted functional magnetic resonance imaging (fMRI) approaches (e.g., Keown et al., 2013; Supekar et al., 2013; Uddin et al., 2013; Di Martino et al., 2014; Nomi and Uddin, 2015). Relatively few studies have investigated structural network changes in children with ASD (e.g., Rudie et al., 2013; Li et al., 2014). To our knowledge, none of the structural network studies were focused on early childhood of 2–7 years. Understanding of brain structural networks in ASD in this early developmental period of 2–7 years may shed light on initial structural connectional configurations that can evolve into both hyper- and hypo-connectivity organizations across different developmental stages and provide a structural basis for functional network alterations.

In this study, we aimed to characterize age-related brain structural network changes in young children with ASD from 2 to 7 years of age, and to identify sensitive network-based imaging biomarkers that are significantly correlated with the symptom severity during this early developmental period. Based on our previous findings of flattened WM microstructural changes quantified by dMRI microstructural measures in children with ASD from the same age range (Ouyang et al., 2016), we hypothesized aberrant hyper-connectivity and slower maturation of structural networks in children with ASD during this same age period. Diffusion MRI was acquired to map the brain structural connectome in 30 children with ASD and 21 TD children. All brain structural networks were constructed with dMRI tractography. We compared graph-theory-based network measurements between TD and ASD individuals and delineated age-related characteristics and alterations of these network properties. We also conducted correlations between graph-theory-based network measurements and symptom severity in children with ASD.

## Materials and Methods

### Children With Autism Spectrum Disorder and Children With Typical Development

All participants were children recruited at Beijing’s Children’s Hospital. The study was approved by the institute Research Ethics Committee, and informed/parental consent was obtained. Thirty male children with ASD aged 2.33–7.00 years (4.15 ± 1.42 years) and 21 male TD children aged 1.99–5.96 years (3.90 ± 1.11 years) participated in this study. TD children at the time of MR imaging were referred for seizures with fever (*n* = 9), intermittent headache (*n* = 10), and strabismus (*n* = 2). All TD children had normal neurological examinations documented in their medical records. The exclusion criteria for TD consisted of known nervous system disease or a history of psychiatric, neurodevelopmental, or systemic illness. Children with ASD were not receiving any CNS-active medications before the MRI studies. The diagnosis of ASD was established using the Autism Diagnostic Interview-Revised (ADI-R) (Lord et al., 1994), Childhood Autism Rating Scale (CARS), Clancy Autism Behavior Scale (CABS) (Clancy et al., 1969), and Autism Behavior Checklist (ABC) (Krug et al., 1980), and confirmed based on expert opinion according to Diagnostic and Statistical Manual of Mental Disorder criteria (American Psychiatric Association [APA], 2013). All diagnoses were performed by two experienced pediatricians with 11 and 12 years of experience in clinical neuropsychology, respectively. These clinical assessments were not performed in TD. Detailed demographics and clinical characteristics of participants are provided in Table 1. Thirty children with ASD have been reported in a prior article (Ouyang et al., 2016) focused on atypical WM microstructure in ASD, whereas in this study we examined completely different brain properties (i.e., the brain macrostructural network) using completely different analysis (i.e., graph-theory analysis and network-based statistics) in a mostly overlapped ASD cohort and a larger TD cohort.

**TABLE 1:** Demographics and clinical assessments of participants.

Parameter	Children with ASD (*n* = 30)	Children with TD (*n* = 21)
Age (years) mean ± SD	4.15 ± 1.42	3.90 ± 1.11
Median (min-max)	3.50 (2.33–7.00)	3.84 (1.99–5.96)
Gender (male/female)	30/0	21/0
*Clinical assessment score*		
Autism behavior checklist (ABC)		
Total score	94.39 ± 7.41	–
Sensory	8.03 ± 3.03	–
Relating	30.97 ± 4.56	–
Stereotypes and object use	9.81 ± 4.23	–
Language	27.29 ± 2.51	–
Self-help and social	18.23 ± 2.90	–
Autism diagnostic interview (ADI-R)	52.77 ± 6.92	–
Childhood autism rating scale (CARS)	41.03 ± 3.79	–
Clancy autism behavior scale (CABS)	18.03 ± 1.96	–

*Data are presented as mean ± standard deviation. All of the scores are raw values. ASD, autism spectrum disorder. TD, typically developing.*

### MRI Data Acquisition

All children were scanned using a 3T Philips Achieva MR System with sedation. Axial diffusion MRI data was acquired with the anterior-posterior commissure (AC-PC) line parallel to the phase-encoding direction. Single-shot, echo-planar imaging (EPI) sequence was used with Sensitivity Encoding parallel imaging scheme (SENSE, reduction factor = 2.5). Eight-channel SENSE head coil was used. Other imaging parameters were as follows: repetition time (TR) = 7.96 s, echo time (TE) = 83 ms, field of view (FOV) = 256 × 256 mm^2^, imaging matrix = 128 × 128, voxel size = 2 × 2 × 2 mm^3^, slice number = 70 covering the entire brain without a slice gap. Diffusion weighting was encoded along 30 independent directions, and *b*-value was 1,000 s/mm^2^. The acquisition was repeated twice to improve signal-to-noise ratio (SNR), resulting in a scan time of 11.5 min. T1-weighted magnetization-prepared rapid gradient-echo (MPRAGE) image was also acquired. Imaging parameters for MPRAGE were as follows: TR/TE = 8.34/3.83 ms, flip angle = 12^°^, FOV = 256 × 256 × 160 mm^3^, voxel size = 1 × 1 × 1 mm^3^. T1-weighted images (T1w) have superior gray and white matter contrast for cortical parcellation. Visual inspection was conducted for all MRI data by the pediatric radiologists (Y.P. and H.C.), and no apparent motion artifacts were found.

### Data Preprocessing

Diffusion MRI data preprocessing including eddy current and motion correction, tensor fitting, and estimation of diffusion MRI-derived measures was conducted with DTIStudio (Jiang et al., 2006). Specifically, eddy current distortion and head motion were corrected by registering all raw diffusion weighted images (DWIs) to a b0 image using a 12-parameter (affine) automated image registration (AIR) algorithm (Woods et al., 1998; Mori and Tournier, 2014). Few motion artifacts were observed in the dMRI datasets. Head motions in dMRI data were quantified for all subjects using the methods described in the literature (Ouyang et al., 2016) and in Supplementary Material. As shown in Supplementary Figure 1, few motion artifacts were observed in both TD and ASD subjects with DWI volume-by-volume translation less than 0.5 mm and rotation around 0.12 degrees for all subjects. There were no significant differences in translation (*p* = 0.719) and rotation (*p* = 0.227) between ASD and TD groups. There were no significant correlations between age and translation (*p* = 0.672) or rotation (*p* = 0.373) in this cohort, either. Standard tensor fitting was conducted to generate dMRI-derived metrics, including fractional anisotropy (FA).

### Network Construction

The two fundamental elements of a network, nodes and edges, were defined using the following procedures to construct an individual structural network.

#### Network Node Definition

**FIGURE 1:**
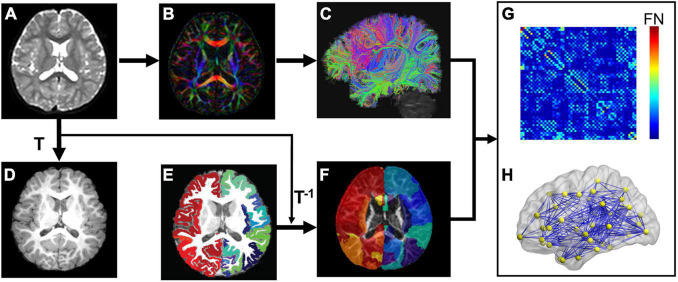
Flowchart of brain white matter (WM) structural network construction. Each subject’s b0 image **(A)** from diffusion magnetic resonance imaging (dMRI) was aligned to the subject’s T1 weighted image (T1w, **D**) with the transformation matrix T. **(B,C)** Shows dMRI tractography results in the subject’s native dMRI space. The subject’s cerebral cortex from T1w was parcellated into 68 regions based on Desikan-Kiliany atlas **(E)**. The cortical ribbon **(E)** was then dilated by 8 mm with in-house program to get through the dense white matter zone for initiating fiber tracking and transferred into the subject’s native dMRI space **(F)** with the inverse transformation of T (T^–1^). With delineation of network edges **(C)** and nodes **(F)** in the native space, connectivity matrix **(G)** and network graph **(H)** were established. The flowchart demonstrates analysis of a representative subject.

Network nodes of each subject in the native dMRI space were obtained by transferring their parcellated cortical regions from their native T1w space (Figures 1A–F). Briefly, contrasts of individual subject’s T1w image (Figure 1D) and b0 image (Figure 1A) in their native dMRI space were used to drive linear registration with a transformation matrix T. T1w image of each subject (Figure 1D) was parcellated into 68 cortical gyri (Figure 1E and Supplementary Table 1) using *Freesurfer* software (Fischl et al., 2004) with Desikan–Kiliany atlas (Desikan et al., 2006). Visual inspection was conducted for *Freesurfer* cortical parcellation by the pediatric radiologists (Y.P. and H.C.) to ensure the parcellation quality as well as to make sure there were no clearly misregistered gyral labels across all subjects in automatic cortical parcellation. The inverse transformation (T^–1^) was used to warp the individual’s cortical parcellations (Figure 1E) into individual native dMRI space. The 68 cortical regions of interest (ROIs) with 34 ROIs in each hemisphere (see Supplementary Table 1 for detail) represented 68 nodes of subject’s structural network (Figure 1). Of note, parcellated cortical ribbon in the dMRI space was dilated by 8 mm toward the deep WM direction (Figure 1F), using an in-house code to penetrate superficial WM and reach deep WM to initiate fiber tracking (Jeon et al., 2015). All registrations were performed using the SPM8 package^1^.

#### Network Edge Definition

Network edge was defined as the number of fiber streamlines connecting two regions (Figure 1F). A brain structural network (Figure 1H) was constructed for each participant, represented by a symmetric 68 × 68 connectivity matrix (Figure 1G). Network edges were defined with reconstructed whole brain white matter fibers. Whole-brain tractography was performed within a brain mask derived from the subject’s b0 image, using diffusion MRI Brute-force deterministic fiber tractography (Mori et al., 1999; Huang et al., 2004) in Diffusion Toolkit.^2^ In this approach, a streamline was initiated from each voxel of the brain mask. Due to relatively low FA in brains of young children, FA threshold was set to 0.15 and angle threshold was 40^°^ for tractography (e.g., Zhao et al., 2019). Only reconstructed fibers with two end points located in the pair of dilated cortical regions, network nodes (Figure 1F), were kept to define network edges (Figure 1C). Number of fiber streamlines (FN) connecting two regions was defined as edge weight (*w*_ij_). To remove spurious connections, we used a minimum threshold of fiber streamlines number (*w*_ij_ = 5) between two regions. As a result, we constructed a weighted structural network (Figure 1H) for each subject, represented by a symmetric 68 × 68 connectivity matrix (Figure 1G).

### Network Analysis

A brain structural network graph *G* (Figure 1H) is composed by *N* nodes and *K* edges. Both global and regional network measures were calculated to provide a summarized scalar for topological characterization of individual brain structural network. Both global and regional network measures were quantified with graph-theory approaches. These network measures characterize the topological organization of brain structural network. Specifically, the following global graph measures were calculated: network strength, global and local efficiency (*Eg* and *Eloc*), and shortest path length (*Lp*) (Rubinov and Sporns, 2010). For regional properties, we calculated nodal efficiency of each node (Rubinov and Sporns, 2010). Based on nodal efficiency, we identified network hubs in both groups, as a node with efficiency that was at least 1 standard deviation larger than averaged nodal efficiency across all nodes. All network analysis was performed using GRETNA software (Wang et al., 2015), and results were visualized using BrainNet Viewer software (Xia et al., 2013). Detailed definitions of these network properties, including network strength, global and local efficiency (*Eg* and *Eloc*), shortest path length (*Lp*) (Rubinov and Sporns, 2010), nodal efficiency *E*_*nodal*_, and hub, are described in Supplementary Material.

### Statistical Analysis

#### Network-Based Statistic

To localize specific altered structural network edges strength in ASD, we used a network-based statistic (NBS)^3^ approach (Zalesky et al., 2012) with age as covariate to identify between-group differences in pairwise edge (or connection). The NBS analysis was performed in three steps. First, a threshold of *p* < 0.05 (before correction) was used to yield *t* statistic (two-sample *t*-tests) matrix of suprathreshold connections, among which any connected components and their size (the number of connections) were determined. Second, a nonparametric permutation approach (10,000 permutations) was used to estimate statistical significance of observed component sizes in the un-corrected connection matrix, controlling family wise error. Briefly, in each permutation, all participants were randomly shuffled into two groups, and two-sample *t*-tests were recomputed to examine group differences in network edges after controlling for age. The same primary threshold (*p* < 0.05) was used to produce suprathreshold connections among which the size of the maximal connected component was recorded. This permutation approach derived the empirical null distribution of connected component size for estimating the significance of observed component sizes. Finally, for a connected component of size N found in the real grouping of control and ASD, its corrected *p*-value was determined by finding the proportion of the 10,000 permutations for which the maximal connected component was larger than N. Interconnected subnetwork components with a corrected *p* < 0.05 were considered statistically significant.

#### Between-Group Differences

To examine between-group difference in global and nodal network properties, a general linear model (GLM) was performed with age as covariate.

#### Age-by-Group Interaction

To assess age-related alteration in global network properties and nodal efficiency, the age-by-group interaction term was added into GLM as the main effect with age and group as covariates. If interaction effect was significant, rates of age-dependent trendline in network measures would be significantly different between groups.

#### Correlation With Clinical Assessments

For the network nodes or edges with significant group differences, relationships between network properties and clinical assessments in ASD were explored using GLM with network measures as dependent variables and clinical assessments (Table 1) as independent variables. Subject age was treated as confounding covariate in the GLM models.

All GLM analyses were performed in *R statistic-software* (version 3.5.1).^4^ For global network measures, a *p* < 0.05 was considered significant. For regional network metrics, multiple comparisons were corrected with false discovery rate correction within each hemisphere (*p* < 0.05). To capture more subtle differences in group comparisons of regional network metrics, we used a less strict false-discovery rate (FDR) correction for multiple comparisons within a hemisphere instead of the whole brain.

## Results

The demographic and clinical assessment data for children with ASD and TD are shown in Table 1. There was no significant difference in median age between ASD and TD (Wilcoxon rank-sum test, *p* = 0.8). Assessed clinical scores of 30 children with ASD ranged from 79 to 107 (mean ± sd: 94.39 ± 7.41) for total score of ABC scale, 41–67 (52.77 ± 6.92) for ADI-R, 36–50 (41.03 ± 3.79) for CARS, and 14–22 (18.03 ± 1.96) for CABS (Table 1).

### Flattened Age-Related Characteristics of Structural Network in Autism Spectrum Disorder at Whole-Brain, Global Level

**FIGURE 2:**
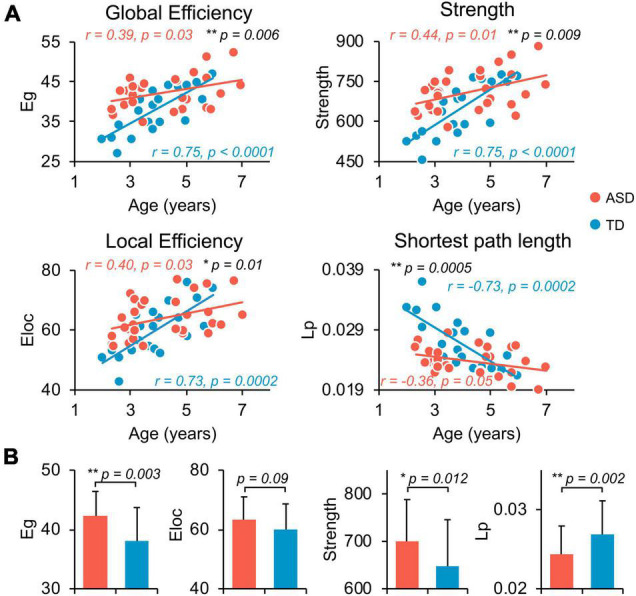
Flattened age-related characteristics of structural network in ASD at whole-brain, global level. **(A)** Scatter plots show the age-dependent trendlines of global measures for ASD (red) and TD (blue) groups. Significant age × group interaction effects were found for all global measures (all *p* ≤ 0.01 in black). *Eg*, global efficiency; *Eloc*, local efficiency; *Lp*, shortest path length. * 0.01 ≤ *p* < 0.05; ** *p* < 0.01. **(B)** Bar charts show the group differences in global network measures between ASD (red) and TD (blue) groups after removing the effect of age. Bars and error bars represent the fitted values and standard deviations, respectively.

Both TD and ASD groups showed significantly age-related increases in global efficiency (TD: *r* = 0.75, *p* < 0.0001; ASD: *r* = 0.39, *p* = 0.03), local efficiency (TD: *r* = 0.73, *p* = 0.0002; ASD: *r* = 0.40, *p* = 0.03), network strength (TD: *r* = 0.75, *p* < 0.0001; ASD: *r* = 0.44, *p* = 0.01), and decrease in network shortest path length (TD: *r* = −0.73, *p* = 0.0002; ASD: *r* = −0.36, *p* = 0.05) from 2 to 7 years old (Figure 2A). However, the growth rates of global network measures were significantly slower in ASD compared with TD, revealed by age-by-group interaction analyses (Figure 2A, global efficiency *p* = 0.006, network strength *p* = 0.009, local efficiency *p* = 0.01, and shortest path length *p* = 0.0005). Overall, from 2 to 7 years of age, children with ASD exhibited a significant hyper-connectivity pattern across multiple global network measures, including increased network strength (*t* = 2.597, *p* = 0.012), increased global efficiency (*t* = 3.161, *p* = 0.003), and decreased shortest path length (Lp, *t* = −3.34, *p* = 0.002) (Figure 2B). We also investigated motion effects on age-by-group interaction and group comparison results at the global level and found that the above findings were not affected after statistically controlling for motion estimates in dMRI scans in the statistical analyses, as shown in Supplementary Tables 2, 3.

### Flattened Age-Related Trends of Nodal Efficiency in Structural Network of Autism Spectrum Disorder at Regional Level

**FIGURE 3:**
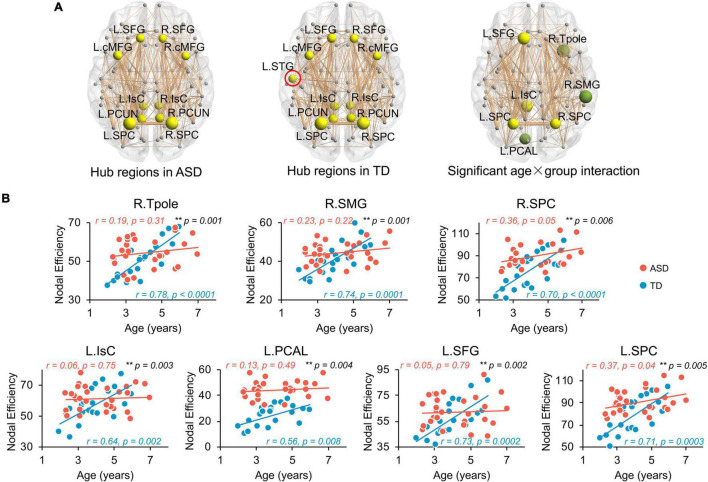
Flattened age-related trends of nodal efficiency in structural network of ASD at regional level. **(A)** Distribution of hub regions of the structural networks. Three dimensional representations of hub region distributions in ASD (left panel) and TD (central panel) groups. Hub nodes are shown in yellow with node sizes indicating their nodal efficiency values and mapped onto a cortical surface at the axial views. The red circle indicates the hub found in TD but not ASD group. Right panel shows the nodes (hub regions in yellow, non-hub region in green) with a significant age × group (TD and ASD groups) interaction in nodal efficiency in a 3D representation of structural network, with node size indicating the significance of interaction. Networks shown here were constructed by averaging WM connection matrices of all subjects in each group at a sparsity of 15%. Network nodes are located according to their centroid stereotaxic coordinates. Network edge widths represent connection strengths between nodes. **(B)** Scatter plots show the age-related trendlines in nodal efficiencies for ASD (red) and TD (blue) groups (all age × group interaction *p* ≤ 0.01 in black). * 0.01 ≤ *p* < 0.05; ** *p* < 0.01. *L*, left hemisphere, *R*, right hemisphere. *BSTS*, banks superior temporal sulcus, *cACC*, caudal anterior cingulate cortex, *cMFG*, caudal middle frontal gyrus, *IsC*, isthmus cingulate cortex, *PCAL*, pericalcarine cortex, *PCC*, posterior cingulate cortex, *PCUN*, precuneus cortex, *PHG*, parahippocampal gyrus, *PrCG*, precentral gyrus, *rMFG*, rostral middle frontal gyrus, *SFG*, superior frontal gyrus, *SMG*, supramarginal gyrus, *SPC*, superior parietal cortex, *STG*, superior temporal gyrus, *Tpole*, temporal pole.

Network hub regions, serving pivotal roles for communication between any pairs of network nodes, were identified for both ASD and TD groups (Figure 3A, left and central panels). Similar hub distributions, with core regions mainly in the frontal and parietal cortices, were found for both groups. Specifically, the bilateral caudal middle frontal gyrus (cMFG), Isthmus cingulate cortex (IsC), precuneus cortex (PCUN), superior frontal gyrus (SFG), and superior parietal cortex (SPC) are common hubs for both groups. The left superior temporal gyrus (STG) was identified as hub only in TD children.

Similar to global network properties, significant age-by-group interactions were found in nodal efficiency across seven brain regions (Figure 3A, right panel, all *p* < 0.006), including bilateral SPC, left IsC, left SFG, left PCAL, right supramarginal gyrus (SMG), and right temporal pole (Tpole). Four regions were network hubs (i.e., bilateral SPC, left IsC, and left SFG) and displayed as yellow nodes in Figure 3A (right panel). Scatter plots clearly show initial high nodal efficiencies in children with ASD, with subsequent flattened and slower development. Nodal efficiencies of structural networks in TD children significantly increased with age from 2 to 7 years old (Figure 3B, all *p* < 0.008). However, relatively flattened age-dependent trendlines of nodal efficiency were observed in ASD, indicating an atypical slower maturation of the structural network based on nodal efficiency. The correlation *r* and *p*-values from linear regression between age and nodal efficiency for both groups are provided in Figure 3B. Similarly, Supplementary Table 3 demonstrates that motion did not contaminate the significant age-by-group interaction in nodal efficiency found in this study.

### Hyper-Connectivity of Structural Network of Autism Spectrum Disorder at Regional Level

**FIGURE 4:**
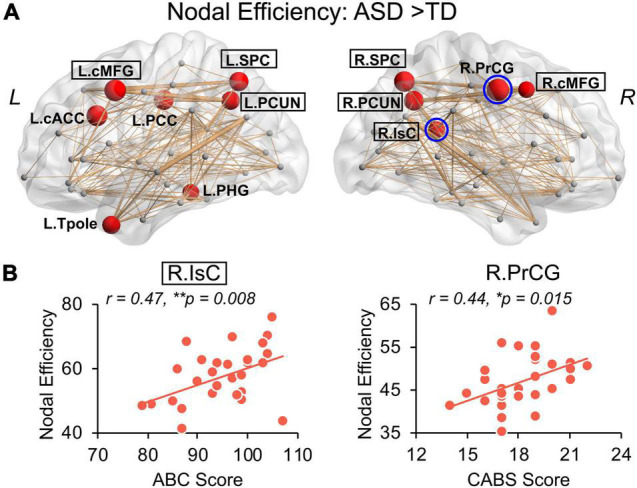
Hyper-connectivity in children with ASD of nodal efficiency correlated with symptom severity. **(A)** Distribution of brain regions, left (*L*) and right (*R*), with significantly higher nodal efficiency in children with ASD after removing age effect. Regions with significant group difference (*p* < 0.05, false discovery rate corrected within each hemisphere) were colored red with node size indicating the significance of between-group differences in the nodal efficiency. Networks shown here were constructed by averaging WM connection matrices of all children with ASD at a sparsity of 15%. Regions with a black box are identified network hubs. **(B)** Clinical correlations with altered nodal efficiency. Scatter plots show the significant positive correlations (*p* < 0.05) between nodal efficiencies from blue circled nodes in **(A)** and total score of Autism Behavior Checklist (ABC) and Clancy Autism Behavior Scale (CABS), respectively. * 0.01 ≤ *p* < 0.05; **p < 0.01. See legend of Figure 3 for abbreviations of brain regions.

Children with ASD also exhibit a hyper-connective pattern in structural networks at the regional level. This hyper-connective pattern is reflected by higher nodal efficiencies in ASD and involves multiple brain subnetworks across several network hub regions (Figure 4A). Significantly higher nodal efficiencies were found in 7 hub regions (i.e., bilateral cMFG, bilateral SPC, bilateral PCUN, and right IsC) and 5 non-hub regions [i.e., left caudal anterior cingulate cortex (cACC), left posterior cingulate cortex (PCC), left parahippocampal gyrus (PHG), left Tpole, and right precentral gyrus (PrCG)] (Figure 4A and Table 2, corrected *p* < 0.05, and Supplementary Table 2 after motion adjustment). Notably, among these hyper-connective regions, bilateral cMFG, bilateral PCUN, left PCC, and right IsC are key regions of functional default-mode network. Left cACC and right PrCG are critical regions of frontoparietal and sensorimotor networks, respectively.

**TABLE 2:** Brain regions with significant group difference between TD and ASD in nodal efficiency.

Regions	Category	*E*_*nodal*_ (mean ± SD)		*t*-value	*p*-value (FDR corrected)
		Children with TD	Children with ASD		
L.cMFG	Hub	55.81 ± 9.93	65.84 ± 8.58	−3.838	0.0003
L.cACC	Non-hub	39.85 ± 8.02	47.51 ± 6.68	−3.710	0.0005
L.PCC	Non-hub	49.90 ± 8.37	57.98 ± 8.00	−3.533	0.0009
L.SPC	Hub	77.90 ± 15.89	90.28 ± 10.56	−3.400	0.0013
L.PCUN	Hub	57.20 ± 9.57	66.10 ± 9.27	−3.264	0.0020
L.Tpole	Non-hub	46.68 ± 8.91	54.98 ± 8.85	−3.196	0.0025
L.PHG	Non-hub	41.61 ± 6.27	47.01 ± 6.38	−2.949	0.0049
R.PrCG	Non-hub	37.29 ± 7.70	45.14 ± 6.04	−3.957	0.0002
R.SPC	Hub	76.26 ± 15.84	89.44 ± 10.48	−3.651	0.0006
R.PCUN	Hub	56.49 ± 10.00	65.54 ± 7.88	−3.505	0.0010
R.IsC	Hub	51.92 ± 9.54	59.65 ± 8.44	−2.938	0.0051
R.cMFG	Hub	53.75 ± 10.72	61.19 ± 7.15	−2.898	0.0056

*The regions with significant increased nodal efficiency (p < 0.05, corrected) are listed in ascending order by absolute t scores in each hemisphere. L, left hemisphere; R, right hemisphere. Cortical regions were classified into hub and non-hub regions. FDR, false discovery rate. See Supplementary Table 1 for abbreviations of brain regions.*

**FIGURE 5:**
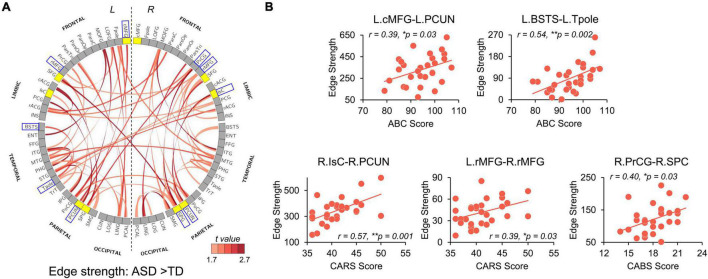
Network Based Statistical (NBS) analysis reveals hyper-connectivity in children with ASD of edge strength that correlated with symptom severity. **(A)** NBS components with significantly higher edge strengths in ASD (*p*-values < 0.05, NBS corrected) are shown in a circle view with the color of edges encoded by the *t*-values from NBS analysis after removing age effect. Yellow nodes indicate hub regions in the structural network of ASD, and gray nodes indicate non-hub regions. **(B)** Scatter plots show significantly positive correlations (*p* < 0.05) between altered edge strengths and clinical scores including ABC, CABS, and Childhood Autism Rating Scale (CARS) scores. These edges were between blue boxed nodes shown in **(A)**. *L*, left hemisphere; *R*, right hemisphere. * 0.01 ≤ *p* < 0.05; **p < 0.01. See Supplementary Table 1 for abbreviations of nodes in **(A)**. See legend of Figure 3 for abbreviations of brain regions.

Significantly higher strength of network edges between widespread brain regions also indicates hyper-connectivity in structural network in ASD (Figure 5). When compared with TD, children with ASD showed significantly increased connection strengths in 61 network edges connecting 51 nodes, widely distributed across the whole brain (Figure 5A, *p* < 0.05 corrected). Higher edge strengths were found in many connections between network hubs demonstrated as yellow nodes in the circle view of Figure 5A, including but not limited to the connections between left cMFG and left PCUN (L.cMFG-L.PCUN), between right IsC and right PCUN (R.IsC-R.PCUN), and between bilateral SPC. Supplementary Figure 2 demonstrates that motion did not contaminate the significantly higher strength of network edges between widespread brain regions in ASD found in this study.

### Hyper-Connectivity in Autism Spectrum Disorder Correlated With Symptom Severity

We further examined the relationship between widely observed hyper-connectivity of structural networks in ASD and symptom severity. Among the 12 nodes with significantly higher nodal efficiency in ASD, two were positively correlated with symptom severity (Figure 4B, *p* < 0.05). Specifically, nodal efficiencies from right IsC of functional default-mode network (*r* = 0.47, *p* = 0.008) and right PrCG of sensorimotor network (*r* = 0.44, *p* = 0.015) in ASD were significantly correlated with the ABC and CABS scores, respectively. In addition, five connections with higher edge strength in ASD were positively correlated with their symptom severity (Figure 5B, *p* < 0.05). Particularly, L.cMFG-L.PCUN (*r* = 0.39, *p* = 0.03) and R.IsC-R.PCUN (*r* = 0.57, *p* = 0.001), connections within functional default-mode network, significantly correlated with ABC and CARS scores, respectively. Connection between left and right rostral middle frontal gyrus (L.rMFG-R.rMFG), as part of frontoparietal network, was significantly correlated with CARS score (*r* = 0.39, *p* = 0.03). Other sensorimotor network related connections between right PrCG and right SPC (R.PrCG-R.SPC; *r* = 0.40, *p* = 0.03), and between left banks superior temporal sulcus (BSTS) and the left Tpole (L.BSTS-L.Tpole; *r* = 0.54, *p* = 0.002) were significantly correlated with ABC and CABS scores, respectively. These findings between clinical scores and nodal efficiency or edge strength demonstrate the significant association between hyper-connectivity from aberrant structural network of ASD and severity of symptoms.

## Discussion

The present study found flattened age-related structural network changes associated with ASD in children from 2 to 7 years of age and revealed a significant correlation between structural network alterations and symptom severity. It sheds light on the brain structural organizational alterations in children with ASD in this critical and rarely studied developmental age of 2–7 years, immediately after the earliest possible ASD diagnosis based on current clinical diagnostic methods. Structural hyper-connectivity in the pivotal default-mode, frontoparietal, and sensorimotor networks underlies the flattened and slower maturation of these structural networks in ASD. Consistent with functions of the default-mode, frontoparietal, and sensorimotor networks, hyper-connectivity in these networks was significantly correlated with quantified symptom severity based on behavioral assessment, complementing the existing structure-function-behavior framework for ASD in early childhood. These network-based hyperconnectivity in ASD over the studied age range may serve as biomarkers potentially predicting the disorder outcomes and setting the stage for possible early intervention.

### Flattened Age-Related Structural Network Changes and Hyper-Connectivity in Children With Autism Spectrum Disorder

Non-uniform brain connectivity findings across different age-ranges support the need to delineate age-related characteristics of structural networks in ASD. The pattern of widespread hyper-connectivity in networks, with relatively slow age-related progression, corroborates the previous microstructural observation of higher WM integrity at an earlier stage and subsequent slower WM maturation in ASD (Ouyang et al., 2016). During typical development from infancy to childhood, WM maturation is characterized by increased WM FA (Ouyang et al., 2019; Yu et al., 2020) and structural network reconfiguration toward stronger and more efficient connectivity (Huang et al., 2015). This pattern of structural development facilitates neural information integration and transformation across brain regions (Achard and Bullmore, 2007). Structural hyper-connectivity found in 2–7-year-old children with ASD is consistent with a previous structural network study of ASD (Li et al., 2018). Enhanced WM microstructural properties with higher FA values across WM bundles in young children with ASD (e.g., Ben Bashat et al., 2007; Ouyang et al., 2016) may contribute to the observed hyper-connectivity in their structural networks as demonstrated by stronger network edges and higher network efficiencies. The pattern of local or short-range hyperconnectivity has been frequently suggested in the brains of individuals with ASD (e.g., Courchesne and Pierce, 2005; Ouyang et al., 2017), especially in younger age groups (e.g., Rudie and Dapretto, 2013). Such pattern was even observed in neonates with high risk of ASD (e.g., Ciarrusta et al., 2020). However, the progression from hyper-connectivity observed in young childhood ASD to the hypo-connectivity observed in other studies from adolescents and adults with ASD implies a pattern of overall connectivity decrease with development, relative to TD children (e.g., Rudie et al., 2013; Di Martino et al., 2014; Nomi and Uddin, 2015).

Importantly, multiple network hubs exhibited significant hyper-connectivity and atypical network property changes, further indicating the crucial role of network hubs in information transfer and their relative vulnerability in brain disorders (Crossley et al., 2014). Given the evolution from hyper- to hypo-connectivity, it is plausible that these atypical network hubs revert into non-hubs with age. As such, the connectivity state of these hubs may be helpful in anticipating the progression of ASD pathology and symptoms. For instance, the left STG, or primary auditory cortex, was a network hub for TD children, whereas children with ASD did not have a network hub in this location. This finding is supported by prior magnetic encephalography (MEG) studies, which also revealed atypical development of the primary auditory cortex in children with ASD, as evidenced by a characteristic delay in their auditory processing (Roberts et al., 2010). Aberrant age-related trends of topological network properties, indicating an important developmental discontinuity, may serve as a neural signature in young childhood ASD.

The human brain network is reconfigured toward an optimal global balance between information segregation and integration during development (Huang et al., 2015; Cao et al., 2017), resulting in the reorganization of the brain network architecture from a relatively randomized configuration to a well-organized one. When examining global network measures of structural networks, we found that individuals with ASD had higher levels of global efficiency and lower levels of shortest path length. Global efficiency reflects the ability to integrate distributed information between distant brain regions and is highly related to long-range connections. Enhanced structural integrity of long-range WM bundles found in ASD (e.g., Ouyang et al., 2016) may contribute to making the brain networks more globally integrated (Cao et al., 2017). Since a random network usually has a short characteristic path length (Sporns, 2011), the altered global network measures in ASD may suggest a less organized or more random distribution of network connections. This is consistent with previous network findings in children with ASD (e.g., Rudie et al., 2013) as well as the increased randomness of brain oscillations findings in resting-state fMRI of individuals with ASD (Lai et al., 2010). Furthermore, structural networks displayed similar levels of local efficiency across both groups in our study. The local efficiency indicates the network segregation capacity, which facilitates functional specialization. Our findings suggest that the network integration and segregation in children with ASD are not appropriately balanced during development.

### Correlation of Hyper-Connectivity in Autism Spectrum Disorder With Symptom Severity

We established the relationship between severe ASD symptoms and greater structural connectivity. This is consistent with prior functional network research that discovered associations between aberrant functional hyper-connectivity and symptom severity in children with ASD (Keown et al., 2013; Lynch et al., 2013; Supekar et al., 2013). Notably, structural hyper-connectivity, which was significantly associated with symptom severity in the present study, was found in regions or connections in functional default-mode, frontoparietal, and sensorimotor networks. Specifically, IsC, cMFG, and PCUN in Figures 4B, 5B are critical hubs of the default-mode network. The default-mode network is considered to play a critical role in self-referential and high-order social cognitive processes, such as Theory of Mind (Buckner et al., 2008). Impairments in this type of mental process are part of the social-cognitive symptoms that characterize ASD (Castelli et al., 2002). Existing research indicates that disruptions in the default-mode network might significantly contribute to social deficits in ASD across the age span (e.g., Monk et al., 2009; Assaf et al., 2010; Lynch et al., 2013; Uddin et al., 2013; Yerys et al., 2015; Padmanabhan et al., 2017). For instance, functional hyper-connectivity in IsC and PCC predicted social communication deficits in children with ASD aged 7–12 years (Lynch et al., 2013), and abnormal PCUN and cMFG functional connectivity significantly correlated with the severity of social and communication deficits in adolescents with ASD aged 11–20 years (Assaf et al., 2010). Abnormal gray matter volumes potentially related to their symptom severity (Uddin et al., 2011) in default-mode network areas were also reported in children and adolescents with ASD. The association in Figure 5B between symptom severity and the banks of superior temporal sulcus (STS) connection might be attributed to social deficits in ASD as well. STS, a multimodal association region involved in cortical integration of both sensory and limbic information, has been recognized as a key cortical area of the social brain (Adolphs, 2003). Anatomical and functional abnormalities in the STS are highly implicated in ASD with social-interaction impairment (Zilbovicius et al., 2006). The observed correlation between structural hyper-connectivity in the frontoparietal network with symptom severity in Figure 5B may be related to executive dysfunction in the ASD population. The frontoparietal network, also known as the central executive network, is involved in highly adaptive cognitive control processes and is critical to the completion of executive functions (Cole et al., 2014). A meta-analysis of fMRI studies on executive functions in ASD aged 7–52 years revealed that abnormal functional connectivity in the frontoparietal network may underline some executive dysfunctions such as rigidity, preservation, and repeated behaviors, which are commonly seen in ASD (May and Kana, 2020). Lastly, identified regions and connections from the sensorimotor network in Figures 4B, 5B are likely due to the motor abnormality in ASD. Previous studies have demonstrated that motor impairments linked with ASD can be observed as early as infancy (Landa and Garrett-Mayer, 2006) and are prevalent in children with ASD (Jansiewicz et al., 2006). Disruption of functional organization within PrCG, a key component of sensorimotor network, was related to ASD diagnosis and to the severity of ASD traits in children (Nebel et al., 2014). Taken together, our findings further elucidate the structural connectional basis for these established functional alterations and revealed the relationship between altered structural connectivity and the phenotype of behavioral impairments in ASD.

### Limitations and Future Directions

We are aware of a few limitations for our study. First, dMRI-based deterministic tractography was adopted to construct network edges. There is potential loss of tracing existing fibers in areas with fiber crossings. We have considered different tractography algorithms in the construction of structural networks in the aspects of connectome specificity and sensitivity. Although deterministic methods yield sparse connectomes with false negatives, more sophisticated probabilistic methods (e.g., Behrens et al., 2007) yield dense connectomes with low specificity due to false positives (Zalesky et al., 2016). Given that only single shell dMRI data with a *b*-value of 1,000 s/mm^2^ along 30 directions was acquired, we adopted deterministic tractography in the current study. Since the same deterministic tractography was applied to all subjects, false negatives in connectomes across subjects were offset as relative connectome metric changes instead of absolute connectome measures were focused on in this study. Second, Freesurfer parcellation of individual T1w images was used to define network nodes. Topological organization of brain networks could be affected by different parcellation strategies when defining network nodes (Zalesky et al., 2010). Despite that choice of network nodes varying across studies, Freesurfer parcellation has been widely used in previous brain network studies (e.g., Dennis et al., 2013) and is able to reliably define brain anatomic regions. Future studies with high-spatial-resolution parcellation strategies in network analysis may further evaluate the reproducibility of our findings. Third, we recognize that atypical development of structural networks in ASD comes from cross-sectional datasets. Future longitudinal studies are warranted to confirm these findings and to fully characterize developmental trajectories of structural network in ASD. Fourth, we only examined the cortico-cortical structural connectivity but not the cortical-subcortical connectivity, such as thalamocortical connectivity. Given the critical role of the thalamus in information processing and cortical functioning of the brain (Sherman, 2016), more research focusing on systematically examining thalamocortical connections is necessary for a more comprehensive understanding of brain connectivity patterns in young children with ASD (e.g., Nair et al., 2021). In addition, considering ASD is more prevalent in males within a general population (Loomes et al., 2017), current study only involved male subjects. Although statistical significance was detected in this study, we acknowledge that larger sample sizes with more homogeneous age distribution and balanced male-to-female ratio are needed for better characterizing such a heterogeneous neurodevelopmental disorder. This work focused on young children aged 2–7 years can also be extended in the future to infants at risk for ASD under 2 years of age for identifying sensitive imaging markers that can predict ASD diagnosis and allowing early intervention at a time prior to the age of diagnosis (Hazlett et al., 2017; Ouyang et al., 2020). Specifically, changes in the brain have been observed in infants at risk for ASD under 2 years of age (e.g., Wolff et al., 2012; Hazlett et al., 2017), preceding clinical manifestations. The changes in brain were also reported for infants under 2 years of age later developing ASD and identified through retrospective study (e.g., Levman et al., 2018, 2019; Shiohama et al., 2021). A machine learning model that leverages sensitive imaging biomarkers may be capable of predicting the clinical diagnostic outcome of individual infants before the development of the full syndrome. The prediction makes early detection and intervention possible and has a significant potential impact on improving outcomes.

## Conclusion

In conclusion, our results suggest that hyper-connectivity with slower subsequent maturation of structural networks is a key component of the underlying neurobiology of young childhood ASD. These networks with hyper-connectivity are significantly correlated with ASD symptom severity in the 2–7-year-old range. Measurements of structural connectivity as indicated by network efficiency and edge strength may serve as early predictors of abnormal developmental trajectories, thereby designating patients with a need for more targeted early intervention.

## Data Availability Statement

The original contributions presented in the study are included in the article/Supplementary Material. The MRI data is available from the website http://brainmrimap.org, a public website maintained by Huang lab, or upon reasonable request to the corresponding author.

## Ethics Statement

This study involving human participants was reviewed and approved by the Beijing Children’s Hospital, Capital Medical University. Written informed consent to participate in this study was provided by the participants’ legal guardian/next of kin.

## Author Contributions

HH and YP designed the study. MO, YP, SS, DH, TZ, HC, and HH performed the research and wrote the manuscript. MO analyzed the data. All authors contributed to the article and approved the submitted version.

## Conflict of Interest

The authors declare that the research was conducted in the absence of any commercial or financial relationships that could be construed as a potential conflict of interest.

## Publisher’s Note

All claims expressed in this article are solely those of the authors and do not necessarily represent those of their affiliated organizations, or those of the publisher, the editors and the reviewers. Any product that may be evaluated in this article, or claim that may be made by its manufacturer, is not guaranteed or endorsed by the publisher.

## Footnotes

1
https://www.fil.ion.ucl.ac.uk/spm/software/spm8/

2
http://trackvis.org/

3
https://nitrc.org/projects/nbs

4
https://r-project.org
