# Supplementary material for: Flattened Structural Network Changes and Association of Hyperconnectivity With Symptom Severity in 2–7-Year-Old Children With Autism
Source: Front Neurosci. 2022 Feb 14;15:757838. doi: 10.3389/fnins.2021.757838 (PMC8882907; doi:10.3389/fnins.2021.757838)
Supplement: Supplementary file 1 [file Data_Sheet_1.docx]

***Supplementary Material***

**Flattened structural network changes and association of hyperconnectivity with symptom severity in** **2-to-7-year-old children with autism**

1. **Supplementary Methods**

*Estimation of the motion in the diffusion MRI scans of children with ASD and TD*

All subjects were sedated during MR scans so that very limited motion artifacts were found in the diffusion MRI (dMRI) datasets. To quantify head motion in each dMRI scan, all diffusion weighted image (DWI) volumes were aligned to the first stable image volume in the scan using automatic image registration in DTIStudio. The volume-by-volume translation and rotation from the rigid registration were calculated using the protocol in the literature (Yendiki et al., 2014). The specific measurements quantifying motions are as follows. 1) *Average volume-by-volume translation:* The translation vector between each pair of consecutive volumes was obtained from the translation component of the rigid registration. Then averaged magnitude of these translation vectors over all DWI volumes in each scan was calculated. 2) *Average volume-*by-volume rotation: The rotation angle between each pair of consecutive volumes was obtained from the rotation component of the rigid registration. Then averaged sum of the absolute values of these rotation angles over all DWI volumes in each scan was calculated.

*Global network organization*

To characterize topological organization of this structure network, following graph measures were calculated: network strength, global and local efficiency (*Eg* and *Eloc*), and shortest path length (*Lp*) (Rubinov and Sporns, 2010). For a weighted brain network *G*, we first calculated the strength of network as:

$$Strength\left( G \right)=\frac{1}{N}\sum_{i\epsilon G} S(i) (1)$$

where $S(i)$ is the sum of network edge weights $w_{ij}$ (FN values) linking to node *i*. The strength of a network is averaged strengths across all nodes in this network.

The path length between any pair of nodes (e.g. nodes *i* and node *j*) is defined as the sum of edge lengths along this path. In a weighted network, the length of each edge is computed as reciprocal of edge weight ($\frac{1}{w_{ij}}$). The shortest path length, $L_{ij}$, is defined as the length of path for nodes *i* and *j* with the shortest length. The shortest path length of a network is computed as:

$$L_{p}\left( G \right)=\frac{1}{N(N-1)}\sum_{i\neq j\in G} L_{ij} (4)$$

where *N* is number of nodes in network. *Lp* of a network quantifies the ability for information propagation in parallel.

The global efficiency (*Eg*) of network *G* can be computed as (Latora and Marchiori, 2001):

$$Eg\left( G \right)=\frac{1}{N\left( N-1 \right)}\sum_{i\neq j\in G} \frac{1}{L_{ij}} (2)$$

where $L_{ij}$ is the shortest path length between node *i* and node *j* in network *G*.

The local efficiency represents how much a network is fault tolerant, showing how efficiency communication is among the first neighbors of node *i* when it is removed. The local efficiency of graph G is defined as:

$$E_{loc}\left( G \right)=\frac{1}{N}\sum_{i\in G} E_{g}\left( G_{i} \right) (3)$$

where $G_{i}$ denotes subgraph composed of the nearest neighbors of node *i.*

*Regional nodal characteristics and hub distribution*

For regional nodal properties, we considered nodal efficiency of each node, which can be computed as:

$$E_{nodal}\left( i \right)=\frac{1}{\left( N-1 \right)}\sum_{i\neq j\in G} \frac{1}{L_{ij}} (5)$$

where $L_{ij}$ is the shortest path length between nodes *i* and *j* in G. $E_{nodal}\left( i \right)$ measures the average shortest path length between a given node *i* and all other nodes in this network. Node *i* was considered a brain hub if $E_{nodal}\left( i \right)$ was at least 1 standard deviation (SD) larger than the averaged nodal efficiency of network (*i.e.* $E_{nodal}\left( i \right)$> mean + SD).

1. **Supplementary Figures and Tables**

**2.1 Supplementary Figures**

Figure S1 shows the histograms of the averaged volume-by-volume translation (0.46 ± 0.07 mm for children with ASD; 0.45 ± 0.10 mm for children with TD) and rotation (0.11 ± 0.038 degrees for children with ASD; 0.12 ± 0.044 degrees for children with TD) measurements from scans of 30 children with ASD and 21 children with ASD. There are no significant differences in translation (p = 0.719) and rotation (p = 0.227) between ASD and TD groups. There are no significant correlations between age and translation (r = 0.06, p = 0.672) or rotation (r = -0.13, p = 0.373) in this cohort, either. Figure S2 shows that after adjusting the age and motion estimates in the Network Based Statistical (NBS) analysis, significantly higher strengths in ASD were still found in 64 network edges connecting 53 nodes (Fig. S2B), which covers the 61 network edges connecting 51 nodes found before the motion estimates were adjusted (Fig. S2A).


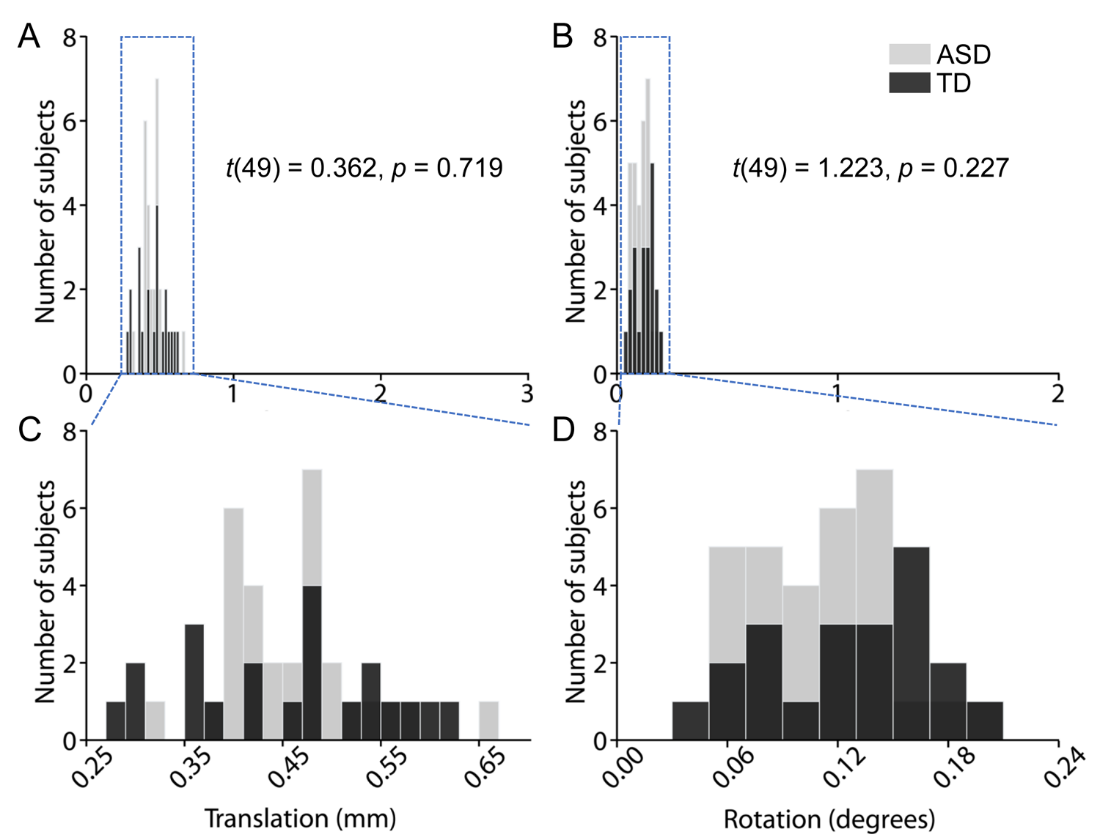


**Figure S1.** Histograms of the averaged volume-by-volume translation measurement and rotation measurement for the scans of 21 children with TD and 30 children with ASD included in this study. No significant differences of translations (*t* (49) = 0.362, *p* = 0.719) or rotations (*t* (49) = 1.223, *p* = 0.227) were found between TD and children with ASD. Panels C and D are enlarged from the dashed boxed in panels A and B, respectively.

**Figure S2.** Network Based Statistical (NBS) analysis reveals hyper-connectivity in children with ASD of edge strength before (A) and after (B) adjustment of motion estimates. (A) NBS components with significantly higher strengths in ASD (p values < 0 .05, NBS corrected) in 61 network edges (blue) connecting 51 nodes, shown on sagittal, axial and coronal views after removing age effects. Black dots indicate the network nodes in the structural network. (B) NBS components with significantly higher strengths in ASD (p values < 0 .05, NBS corrected) in 64 network edges (blue) connecting 53 nodes, shown on sagittal, axial and coronal views after removing age effects and adjustment of motion estimates.

**2.2 Supplementary Tables**

**Table S1.** Cortical regions of interest defined in the study.

| **Abbreviation** | **Region** |
| --- | --- |
| BSTS | Banks superior temporal sulcus |
| cACC | Caudal anterior cingulate cortex |
| cMFG | Caudal middle frontal gyrus |
| CUN | Cuneus cortex |
| ENT | Entorhinal cortex |
| Fpole | Frontal pole |
| FFG | Fusiform gyrus |
| IPC | Inferior parietal cortex |
| ITG | Inferior temporal gyrus |
| IsC | Isthmus cingulate cortex |
| LOC | Lateral occipital cortex |
| LOFC | Lateral orbitofrontal cortex |
| LING | Lingual gyrus |
| MOFC | Medial orbitofrontal cortex |
| MTG | Middle temporal gyrus |
| PHG | Parahippocampal gyrus |
| ParaC | Paracentral lobule |
| ParsOp | Pars opercularis |
| ParsOr | Pars orbitalis |
| ParsTri | Pars tringularis |
| PCAL | Pericalcarine cortex |
| PoCG | Postcentral gyrus |
| PCC | Posterior cingulate cortex |
| PrCG | Precentral gyrus |
| PCUN | Precuneus cortex |
| rACC | Rostral anterior cingulate cortex |
| rMFG | Rostral middle frontal gyrus |
| SFG | Superior frontal gyrus |
| SPC | Superior parietal cortex |
| STG | Superior temporal gyrus |
| SMG | Supramarginal gyrus |
| Tpole | Temporal pole |
| TrT | Transverse temporal cortex |
| INS | Insula |

Note. ─The regions are listed in terms of Desikan-Kiliany atlas (Desikan et al., 2006).

**Table S2.** Motion-adjusted group difference in global network properties and nodal efficiency.

| **Network property** | **Before motion adjustment** | **After motion adjustment** |
| --- | --- | --- |
| *Group difference between TD and ASD in global network properties* | | |
| Global efficiency | t=-3.161, p=0.003** | t=-3.182, p=0.003** |
| Local efficiency | t=-1.739, p=0.09 | t=-2.023, p=0.049* |
| Strength | t=-2.597, p=0.012* | t=-2.784, p=0.008** |
| Shortest path length | t=3.34, p=0.002** | t=3.32, p=0.002** |
| *Group difference between TD and ASD in nodal efficiency* | | |
| L.cMFG | t=-3.838, p = 0.0003** | t=-3.633, p = 0.0007** |
| L.cACC | t=-3.710, p = 0.0005** | t=-3.283, p = 0.0019** |
| L.PCC | t=-3.533, p = 0.0009** | t=-3.766, p = 0.0005** |
| L.SPC | t=-3.400, p = 0.0013** | t=-3.404, p = 0.0013** |
| L.PCUN | t=-3.264, p = 0.0020** | t=-3.345, p = 0.0016** |
| L.Tpole | t=-3.196, p = 0.0025** | t=-3.468, p = 0.0011** |
| L.PHG | t=-2.949, p = 0.0049** | t=-2.849, p = 0.0065** |
| R.PrCG | t=-3.957, p = 0.0002** | t=-3.309, p = 0.0018** |
| R.SPC | t=-3.651, p = 0.0006** | t=-3.672, p = 0.0006** |
| R.PCUN | t=-3.505, p = 0.0010** | t=-3.309, p = 0.0018** |
| R.IsC | t=-2.938, p = 0.0051** | t=-2.749, p = 0.0085** |
| R.cMFG | t=-2.898, p = 0.0056** | t=-2.896, p = 0.0058** |

Note. - L: left hemisphere; R: right hemisphere. * 0.01≤ *p < 0.05*; ** *p < 0.01*. See Table S1 for abbreviations of brain regions.

**Table S3.** Motion-adjusted age-by-group interaction in global network properties and nodal efficiency.

| **Network property** | **Before motion adjustment** | **After motion adjustment** |
| --- | --- | --- |
| *Age-by-group interaction in global network properties* | | |
| Global efficiency | p=0.006** | p=0.009** |
| Local efficiency | p=0.01* | p=0.024* |
| Strength | p=0.009** | p=0.014* |
| Shortest path length | p=0.0005** | p=0.001** |
| *Age-by-group interaction in nodal efficiency* | | |
| L.IsC | p=0.003** | p=0.006** |
| L.PCAL | p=0.004** | p=0.004** |
| L.SFG | p=0.002** | p=0.002** |
| L.SPC | p=0.005** | p=0.008** |
| R.Tople | p=0.001** | p=0.002** |
| R.SMG | p=0.001** | p=0.002** |
| R.SPC | p=0.006** | p=0.010** |

Note. - L: left hemisphere; R: right hemisphere. * 0.01≤ *p < 0.05*; ** *p < 0.01*. See Table S1 for abbreviations of brain regions.

**Supplementary References**

Desikan RS, Ségonne F, Fischl B, et al. An automated labeling system for subdividing the human cerebral cortex on MRI scans into gyral based regions of interest. Neuroimage 2006; 31(3): 968-980. doi:10.1016/j.neuroimage.2006.01.021

Latora V, Marchiori M. Efficient behavior of small-world networks. Phys Rev Lett 2001; 87(19):198701. doi:10.1103/PhysRevLett.87.198701

Rubinov M, Sporns O. Complex network measures of brain connectivity: uses and interpretations. Neuroimage 2010; 52(3): 1059-1069. doi:10.1016/j.neuroimage.2009.10.003

Yendiki A, Koldewyn K, Kakunoori S, Kanwisher N, Fischl B. Spurious group differences due to head motion in a diffusion MRI study. *NeuroImage* 2014; 88:79-90. doi: 10.1016/j.neuroimage.2013.11.027
